# Supplementary figures and images for: Cluster replicability in single-cell and single-nucleus atlases of the mouse brain
Source: bioRxiv. 2025 Feb 25:2025.02.24.639959. Preprint. [Version 1] doi: 10.1101/2025.02.24.639959 (PMC11888248; doi:10.1101/2025.02.24.639959)

Clusters with best-vs-next AUROC > 0.95

Markers used

Dataset

Cells

Nuclei

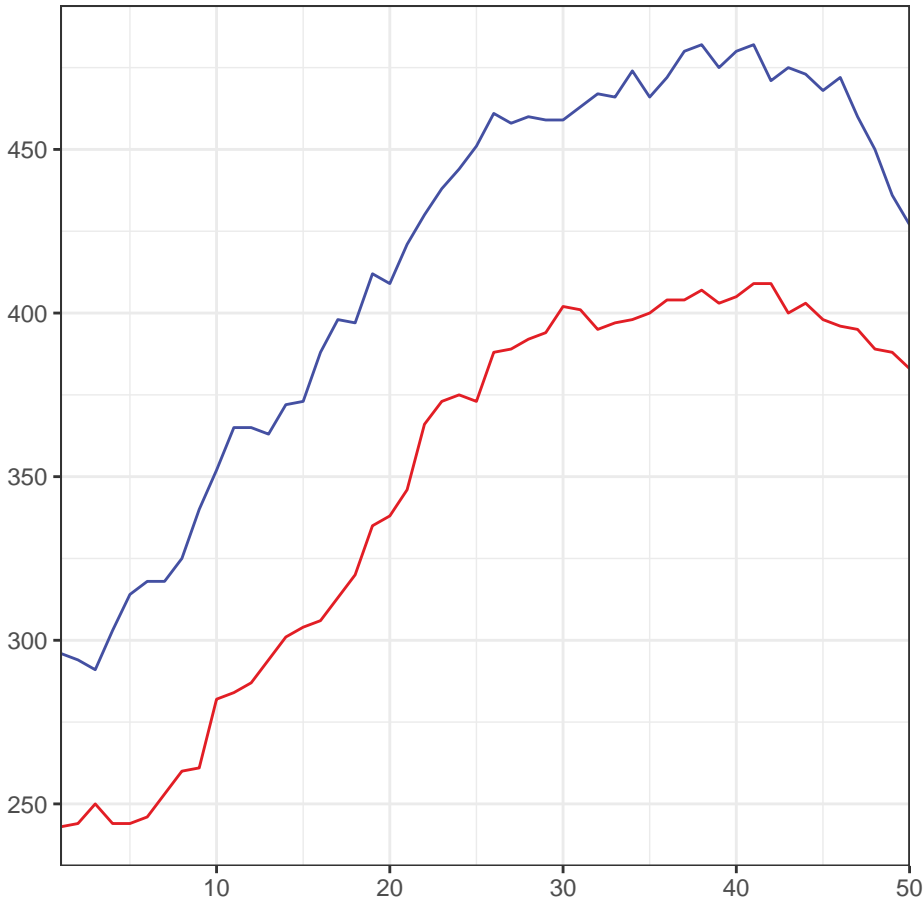

Supplement: Supplement 9 — Supplementary Figure 1: Number of reciprocal best-hit clusters achieving a best-versus-next AUROC of 0.95 or greater (y-axis) for varying numbers of MetaMarkers (x-axis) for single-cell (blue) and single-nuclei (red) datasets. [file media-9.pdf]

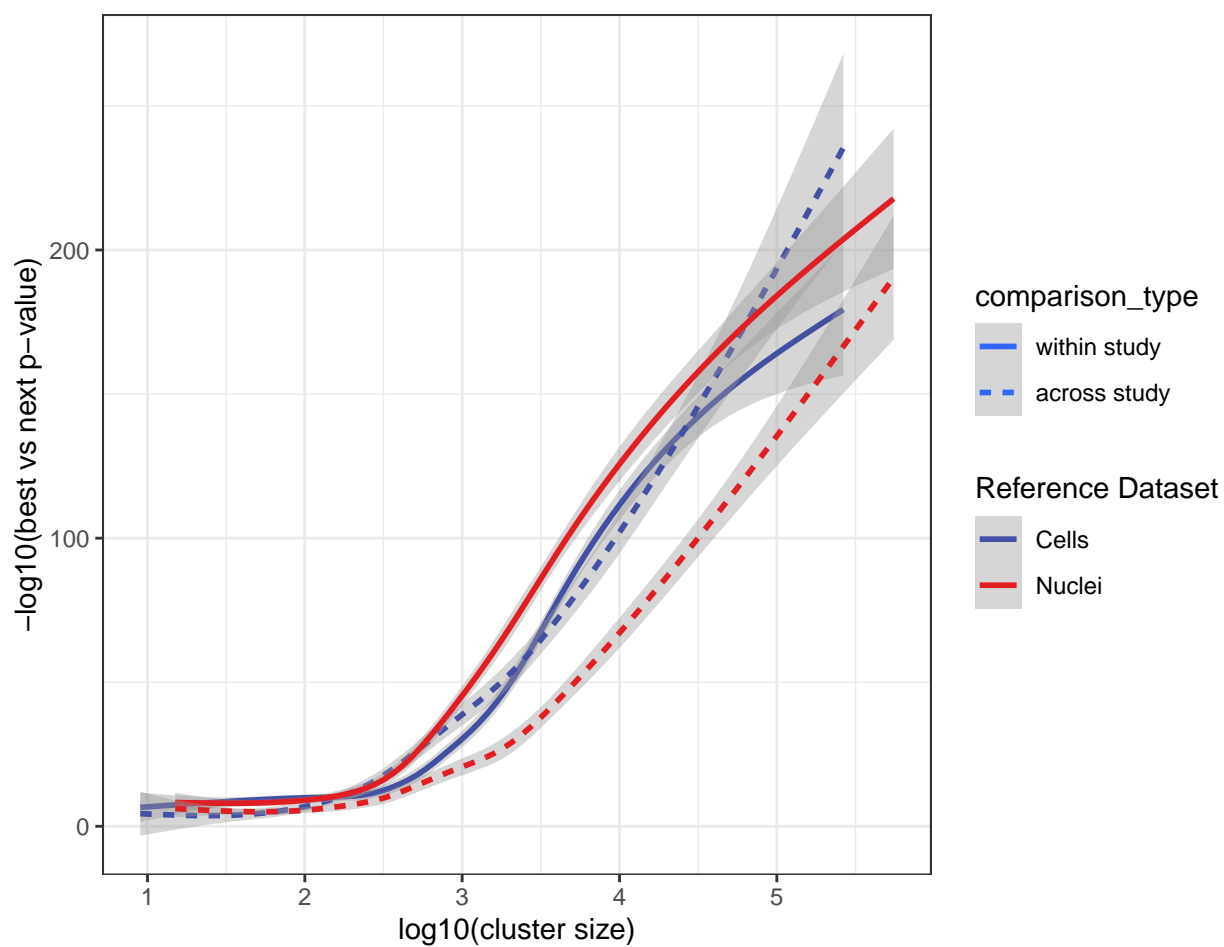

Supplement: Supplement 10 — Supplementary Figure 2: Smoothed trends of discrimination performance (negative log10 Mann–Whitney U test p-value) versus cluster size (log-scaled) for best-versus-next cluster comparisons. The estimated trend lines were computed using Generalized Additive Models (GAMs) via geom_smooth() in R's ggplot2, with shaded areas indicating 95% confidence intervals. Solid lines depict the association within a dataset and dashed lines show it across datasets for reference clusters derived from cells (blue) and nuclei (red). [file media-10.pdf]
